# The Emerging Landscape of Long Non-Coding RNAs in Colorectal Cancer Metastasis

**DOI:** 10.3389/fonc.2021.641343

**Published:** 2021-02-25

**Authors:** Zhiming Liao, Hui Nie, Yutong Wang, Jingjing Luo, Jianhua Zhou, Chunlin Ou

**Affiliations:** ^1^ Department of Pathology, Xiangya Hospital, Central South University, Changsha, China; ^2^ Teaching and Research Room of Biochemistry and Molecular Biology, Medical School of Hunan University of Traditional Chinese Medicine, Changsha, China; ^3^ National Clinical Research Center for Geriatric Disorders, Xiangya Hospital, Central South University, Changsha, China

**Keywords:** colorectal cancer, lncRNAs, cancer metastasis, signaling pathways, markers, therapy

## Abstract

Colorectal cancer (CRC) is one of the most common gastrointestinal cancers, with extremely high rates of morbidity and mortality. The main cause of death in CRC is distant metastasis; it affects patient prognosis and survival and is one of the key challenges in the treatment of CRC. Long non-coding RNAs (lncRNAs) are a group of non-coding RNA molecules with more than 200 nucleotides. Abnormal lncRNA expression is closely related to the occurrence and progression of several diseases, including cancer. Recent studies have shown that numerous lncRNAs play pivotal roles in the CRC metastasis, and reversing the expression of these lncRNAs through artificial means can reduce the malignant phenotype of metastatic CRC to some extent. This review summarizes the major mechanisms of lncRNAs in CRC metastasis and proposes lncRNAs as potential therapeutic targets for CRC and molecular markers for early diagnosis.

## Introduction

Colorectal cancer (CRC) is currently the third most common malignant tumor worldwide. Approximately 1.8 million new cases and nearly 900,000 deaths are reported worldwide each year. The high incidence and high mortality of CRC are serious threats to human health (1, 2). The occurrence and development of CRC is a complex process that involves exogenous and endogenous factors, such as Signaling molecules, homeostasis, microenvironment, diet, and lifestyle, which play an important role in the CRC pathogenesis (3, 4). In recent years, the molecular pathological epidemiology (MPE) has showed that the diet and lifestyle are closely related to the tumorigenesis. For example, smoking, eating red and processed meat, excess alcohol intake, and certain drugs (e.g., aspirin) have been confirmed to be related to the occurrence and development of CRC (5). With the rapid progress in clinical treatment, the 5-year survival rates of patients with CRC has improved significantly. However, the treatment outcomes in patients with metastatic CRC are still not ideal, and the 5-year survival rate in such patients is only ~12% (6–8). Metastasis of CRC is an important factor leading to the CRC recurrence and death. Therefore, elucidating the molecular mechanism of CRC metastasis and identifying molecular markers related to metastasis are critical for improving the treatment outcomes of CRC.

Long non-coding RNAs (lncRNAs) are non-coding RNA molecules that are greater than 200 nucleotides in length. Most of them are transcribed by RNA polymerase II and share similarities with messenger RNAs (mRNAs), although they lack coding ability (9). lncRNAs can be divided into five categories according to their positional relationship with protein-coding genes: sense, antisense, bidirectional, inter-intron, and intergenic lncRNAs (10). Accumulating evidence strongly suggests that lncRNAs are an important class of molecules that regulate genomic processes. The long nucleotide chain of lncRNAs can either form a complex spatial structure and interact with protein factors, or provide a large binding site for the concurrent binding of several molecules that collectively participate in X-chromosome silencing, genomic imprinting, epigenetic regulation, transcriptional activation or interference, nuclear and cytoplasmic trafficking, mRNA splicing and degradation, and genomic imprinting, among others (11). Since lncRNAs play important roles in various aspects of gene expression, the relationship between lncRNAs and tumors has become the focus area of current research. A variety of lncRNAs have been shown to promote or suppress tumorigenesis in different cancers. For instance, Zhuang et al. (12) found that lncRNA GClnc1 promotes the proliferation and invasion of bladder cancer by activating MYC expression. LncRNA PVT1 plays a carcinogenic role in prostate cancer and is a potential diagnostic biomarker (13). In CRC, researchers have found numerous differentially expressed lncRNAs and confirmed their important roles in regulating CRC cell proliferation, apoptosis, invasion, and metastasis as well as sensitivity to radiotherapy and chemotherapy (14). For instance, the HOXB-AS3 peptide encoded by lncRNA HOXB-AS3 has been shown to inhibit the growth of CRC (15). Accumulated evidence indicates that lncRNAs are important markers of CRC metastasis. Yue et al. observed that lncRNA CYTOR can promote the CRC metastasis *via* the Wnt/β-catenin signaling pathway (16). Therefore, lncRNAs are potential therapeutic targets for CRC.

## Characteristics and Roles of lncRNAs

Generally, non-coding RNAs can be divided into long-chain and short-chain non-coding RNAs based on their lengths (17). The first long non-coding RNA transcript sequence discovered in eukaryotes has a length of more than 200 nt and an mRNA-like structure. After splicing, a 7mC cap is usually added at the 5′end of the lncRNA sequence, and a polyA tail is sometimes added to the 3′end (18, 19). Studies have shown that for some lncRNAs, corresponding DNA regions are located between genes or introns, some overlap with protein-coding genes, while some lncRNAs encode a small number of functional short peptides (20, 21). While the primary structure of an lncRNA is its nucleotide sequence, its functional activity depends on base pairing but it is less conserved than its higher-order structure (22, 23). The secondary and tertiary structures of lncRNAs determine their functions. The secondary structures mainly include double helices and hairpins, whereas the tertiary structures are more diverse, such as sarcin-ricin loops. The lower conservation of its primary structure is balanced by these higher-order structures (24–26).

The main modes of action reported for lncRNAs include: ① interfering with mRNA cleavage by forming complementary double-stranded RNA (27), ② altering the activity of a specific protein through direct binding (28), ③ changing the cytoplasmic localization of a specific protein through direct binding (29), ④ altering the expression of target genes by inhibiting RNA polymerase II, or through chromatin remodeling and histone modification (30), ⑤ interfering with target gene expression by initiating transcription from the promoter region of protein-coding genes (31), ⑥ forming double-stranded RNAs with the transcripts of protein-coding genes and producing endogenous siRNAs through the action of Dicer (32), ⑦ acting as a structural component by forming a nucleic acid-protein complex (33), and ⑧ acting as the precursor of a small RNAs (such as a miRNAs or piRNAs). LncRNAs are mostly expressed in the nucleus and their expression levels are lower compared to those of mRNAs (34). However, lncRNAs are intricately involved in the regulation of various biological activities owing to their tissue-specific expression, and they can also affect disease processes (35). LncRNAs can also regulate the expression of important genes at multiple levels *via* epigenetic regulation and by modulating transcription, post-transcriptional processes, translation, and protein modification either as an initially transcribed RNA or a mature spliced RNA. Moreover, lncRNAs play important roles in physiological processes including development, tissue differentiation, reproduction, and immunity as well as in the formation and development of tumors.

## Mechanism of lncRNA Action in CRC Metastasis

Tumor metastasis is the process wherein malignant cells detach from the primary tumor site and are translocated through the circulatory system to secondary tissues or organs, where they colonize and form secondary tumors (36). Tumor invasion and metastasis are complex, dynamic processes that typically involve changes in the tumor microenvironment, epithelial-mesenchymal transition (EMT), hypoxia, and angiogenesis among other mechanisms (37). Accumulating studies have shown that lncRNAs regulate CRC metastasis mainly by regulating key factors that simultaneously affect multiple signaling pathways that are closely related to tumor metastasis. In other cases, lncRNAs can sponge miRNAs to regulate the expression of target genes. lncRNAs can also bind directly to proteins to induce the protein degradation *via* affecting their phosphorylation or ubiquitination. Tumor invasion and metastasis affect patient prognosis and survival and are important causes of tumor-related death; hence, blocking these processes remains a critical challenge in cancer treatment (38).

### LncRNAs Regulate CRC Metastasis by Regulating Signaling Pathways

Tumor metastasis involves complex regulatory processes and alteration in multiple molecular signaling pathways in the tumor microenvironment (39, 40). Several pathways, including the Wnt/β-catenin (41), PI3K/AKT (42), STAT (43), MAPK (44), and Notch signaling pathways (45) play key roles in the metastasis of different tumors (**Table 1**).

**Table 1:** LncRNAs and their targeting signaling pathways in the regulation of CRC metastasis.

LncRNAs	Dyregulation	Targets	Signaling pathways	Ref.
EPB41L4A-AS1	Up	GTPase	RhoA/ROCK signaling	(46)
STX17-AS1		RhoA		(47)
XIST		RhoA		(48)
CRNDE	Up	β-catenin/TCF4	Wnt/β-catenin signaling pathway	(49)
LINC01354		hnRNP-D		(50)
LINC00675		GSK-3β		(51)
SNHG15		SIRT1		(52)
NEAT1		DDX5		(53)
CASC11		hnRNP-K		(54)
LINC00689	Down	LATS2	Hippo signaling pathway	(55)
CMPK2	Up	FUBP3	c-Myc signaling pathway	(56)
DILC	Down	STAT3	IL-6/STAT3 signaling pathway	(57)
LINC01296	Up	MUC1	PI3K/AKT/mTOR signaling pathway	(58)
LINC00115		PI3K		(59)
SNHG7		GALNT7		(60)
SNHG14		PI3K/AKT		(61)
HOTAIR		FUT6		(62)
ITIH4-AS1	Up	FUS	JAK/STAT3 signaling pathway	(63)
HOTAIR		ST6GAL1		(64)
TPT1-AS1		TPT1		(65)
GAPLINC	Up	c-MET	c-MET signaling pathway	(66)
FOXC2-AS1	Up	FOXC2	Ca 2^+^-FAK signaling pathway	(67)
MIR22HG	Down	SMAD2	TGF-β/SMAD signaling pathway	(68)
PVT-1	Up	SMAD4		(69)
CASC9		CPSF3		(70)
SNHG6	Up	UPF1		(71)
LINC00941	Up	SMAD4		(72)
DSCAM-AS1	Up	Notch1	Notch signaling pathway	(73)
HOXD-AS1	Down	HOXD3	MAPK signaling pathway	(74)
H19	Up	RAS		(75)
BANCR		MEK		(76)
CRNDE		hnRNPUL2		(77)
cCSC1	Up	SMO and Gli1	Hedgehog signaling pathway	(78)
LUCAT1	Up	RPL40	p53 signaling pathway	(79)
lnc-GNAT1-1	Down	RKIP	NF-κB signaling pathway	(80)
CCAT2	Up	BOP1	AURKB signaling pathway	(81)

Several studies have reported that the Wnt/β-catenin signaling pathway is closely related to CRC metastasis. Yue et al. (16) observed that lncRNA CYTOR, which is highly expressed in CRC, forms a positive feed forward loop with β-catenin and participates in the regulation of colon cancer metastasis. In this process, cell receptors bind to cytoplasmic β-catenin and block β-catenin phosphorylation catalyzed by casein kinase 1 (CK1), leading to the accumulation of β-catenin and its nuclear transport. Subsequently, the β-catenin/TCF complex activates the expression of cell receptor encoding genes, thereby forming a positive feed forward loop. LncRNA SLCO4A1-AS1 inhibits the interaction of β-catenin with GSKβ, inhibits β-catenin phosphorylation, and improves β-catenin stability, ultimately promoting the proliferation, migration, and invasion of CRC cells (82). Wu et al. (83) showed that lncRNA JMJD2C promotes CRC metastasis by enhancing the β-catenin signaling pathway and participating in the regulation of histone methylation at the MALAT1 promoter. In addition to directly participating in β-catenin signaling pathway transduction, lncRNAs can also play indirect regulatory roles in this signaling pathway. Research has shown that NEAT1 indirectly activates the Wnt/β-catenin signaling pathway through DDX5, and therefore, exerts its carcinogenic effects are mediated by DDX5 (53).

The PI3K/AKT signaling pathway also plays a key role in CRC metastasis, and several lncRNAs have been shown to modulate this pathway. Song et al. (84) found that the expression of the lncRNA, PlncRNA-1, was significantly higher in CRC tissues, and PlncRNA-1 knockout significantly reduced the spread, migration, and invasion of CRC cells. Further functional analysis showed that PlncRNA-1 affects the growth and metastasis of CRC mainly through the PI3K/AKT signaling pathway. The lncRNA SNHG6 inhibits ETS1 expression by directly targeting its 3′-untranslated region (UTR) and inhibiting the expression of phosphoinositide 3-kinase (PI3K)/protein kinase B (AKT)/rapamycin mechanical target (mTOR) to activate the CRC invasion (85). In addition, Wang et al. (86) found that lncRNA AB073614 promotes the proliferation and metastasis of CRC cells mainly through the PI3K/AKT signaling pathway. The lncRNA ST3Gal6 antisense 1 (ST3Gal6-AS1) is derived from the promoter region of gene encoding sialyltransferase ST3Gal6, and it mediates α-2,3 sialylation through the ST3Gal6-AS1/ST3Gal6 axis, thereby regulating PI3K/Akt signaling and leading to the nuclear translocation of Foxo1 in CRC cells (87).

Several other signaling pathways have been confirmed to play important roles in CRC metastasis. Functional analysis has shown that the lncRNA FEZF1-AS1, which is upregulated in CRC tissues, can bind to pyruvate kinase 2 (PKM2) protein and improve its stability. Higher cytoplasmic levels of PKM2 promote pyruvate kinase activity and lactate production (aerobic glycolysis), whereas higher nuclear levels of PKM2, induced by FEZF1-AS1, activate STAT3 signaling, which promotes the proliferation and metastasis of CRC cells (88). Zhou et al. (78) found that lncRNA-cCSC1 can modulate the characteristics of CRC stem cells by activating the Hedgehog signaling pathway and thus, plays an important role in CRC metastasis.

The migration and invasion of tumor cells require cytoskeletal rearrangement. Tang et al. (89) reported that lncRNAs can directly regulate the cytoskeleton in a variety of tumors and can alter the cytoskeleton *via* Rho/ROCK signaling during tumor migration. The lncRNA EPB41L4A-AS1 is overexpressed in CRC tissues and may affect proliferation, invasion, and migration by activating the Rho/ROCK-related protein kinase signaling pathway. Therefore, EPB41L4A-AS1 could be used as a new biomarker for the diagnosis and targeted treatment of CRC (90). Further, Tang et al. (91) studied the specific role of lncRNA-SLCO4A1-AS1 in CRC and found that its effects on cell proliferation, migration, and invasion were mainly associated with regulating the EGFR/MAPK pathway. Studies have shown that 1α, 25-(OH)2D and vitamin D receptor (VDR) in CRC cells stimulate MEG3 expression by directly binding to the promoter of lncRNA MEG3; MEG3 acts as a tumor suppressor by regulating clusterin activity. Therefore, the VDR/lncRNA MEG3/clusterin signaling pathway is a potential therapeutic target and prognostic biomarker for CRC patients (92).

### LncRNAs Regulate CRC Metastasis Through Sponging miRNA

**Figure 1:**
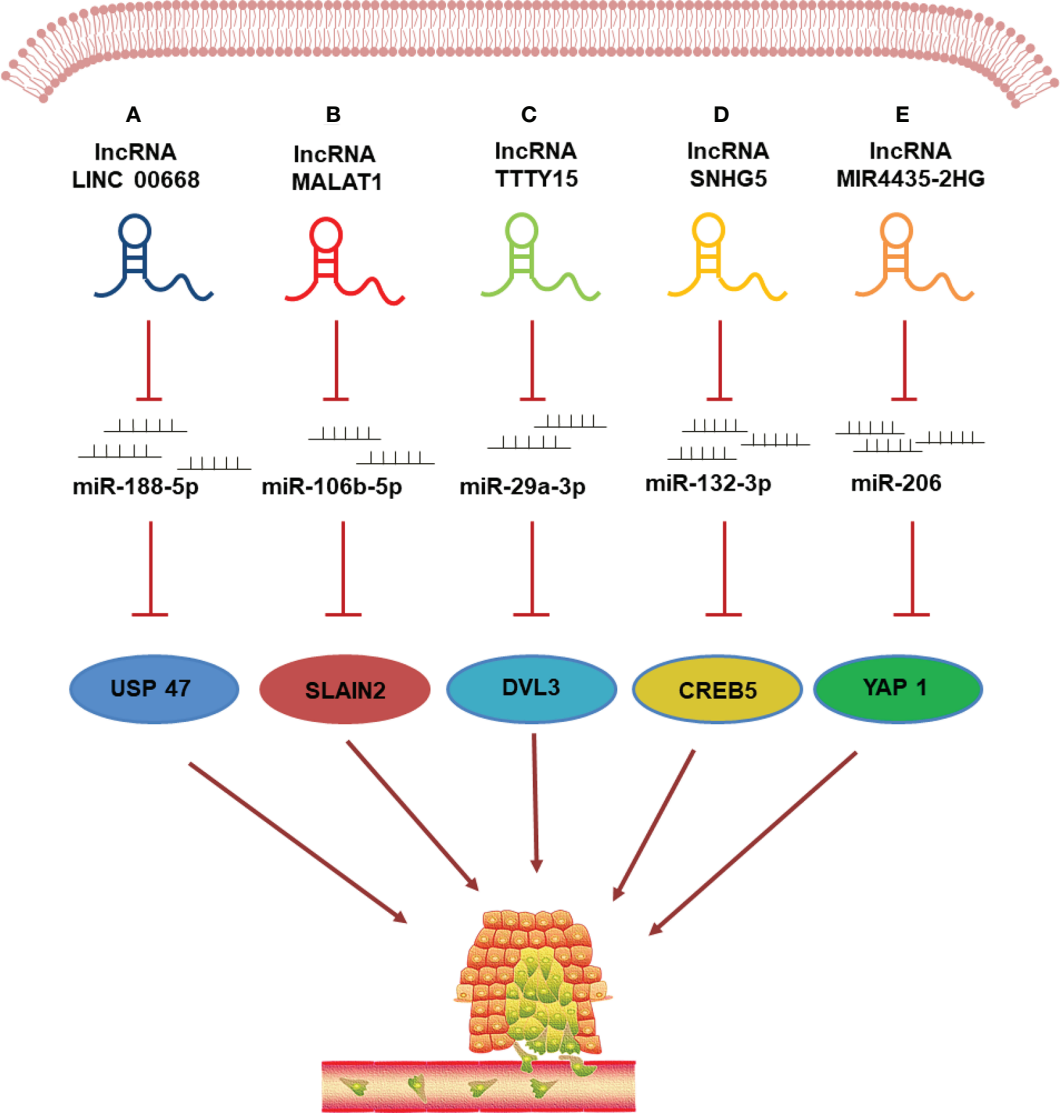
LncRNAs regulate CRC metastasis by sponging miRNAs. **(A)** lncRNA LINC00668 promotes the metastasis and infiltration of CRC cells by sponging miR-188-5p and weakening its inhibiting effect on USP47 expression; **(B)** lncRNA MALAT1 regulates the miR-106b-5p expression by functioning as a competing endogenous RNA (ceRNA) and regulates the SLAIN2-associated microtubule mobility, leading to the CRC progression; **(C)** lncRNA TTTY15 functions as the ceRNA to regulate the expression of target gene DVL3 by sponging miR-29a-3p to promote CRC metastasis; **(D)** lncRNA-SNHG5 influences CRC cell metastasis by modulating the SNHG5/miR-132-3p/CERB5 axis. **(E)** lncRNA MIR4435-2HG acts as a ceRNA to promote the metastasis of CRC *via* upregulating YAP1 expression by sponging miR-206.

In recent years, several studies have shown that since lncRNAs contain several introns, they can sponge miRNAs to form competing endogenous RNA (ceRNA) networks. LncRNAs are transported to target cells *via* circulation, bind to intracellular miRNAs, sponge them, and limit their ability to interfere with the translation of their target mRNAs; a process important for cancer cell proliferation, invasion, migration, and apoptosis. Thus, the ability to sponge miRNAs is an important mechanism by which lncRNAs regulate CRC metastasis (**Figure 1**).

Yan et al. (93) reported the lncRNA LINC00668, which is encoded on chromosome 18p11.31, as a newly discovered lncRNA associated with cancers. LINC00668 is upregulated in CRC cancer tissues and cells and studies have shown that LINC00668 can bind to miR-188-5p in CRC cells. Therefore, LINC00668 may play a carcinogenic role in CRC by sponging miR-188-5p and upregulating USP 47 expression. Shan et al. (94) found that lncRNA SNHG7 regulates GALT1 levels by activating miR-216b and plays a carcinogenic role in CRC development. Xu et al. (95) reported that MIR17HG promotes CRC by inducing NF-κB/RELA expression and competitively sponging miR-375. LncRNA-SNHG5 has been shown to affect the proliferation, metastasis, and migration of CRC cells by regulating miR-132-3p/CREB5 (96). LncRNA-CRNDE modulates CRC progression and chemotherapy resistance by regulating the expression level of miR-181a-5p and the activity of the Wnt/β-catenin signaling pathway (49). LncRNA HNF1A-AS1, which is upregulated in colon cancer tissues, is closely related to clinical staging, vascular invasion, lymph node metastasis, and distant metastasis. In addition, HNF1A-AS1 regulates the expression of miRNA-34a by acting as a ceRNA, thereby inhibiting the miR-34a/SIRT1/p53 feedback loop and activating the Wnt signaling pathway to promote the development of colon cancer (97). LncRNA MIR4435-2HG was first found in lung cancer tissues where it functions as a ceRNA and sponges miR-206 to upregulate the expression of YAP 1. MIR4435-2HG promotes the CRC growth and metastasis *via* the miR-206/YAP 1 axis (98). A functional analysis by Yang et al. (99) showed that knocking out lncRNA-FTX significantly inhibited the proliferation, migration, and invasion of CRC cells. Further analysis showed that FTX could directly interact with miR-215 and inhibit its expression, thereby inhibiting the metastasis of CRC. In CRC cells, the expression of lncRNA TUG1 is abnormally high, whereas the expression of miR-600 is downregulated in CRC tissues, cell lines, and metastatic tissues. Moreover, TUG1 inhibits the migration, invasion, and EMT of CRC cells by competing with miR-600 (100).

Li et al. (101) revealed the previously unrecognized role of the lncRNA ZDHHC8P1/miR-34a regulatory axis in regulating the progression and metastasis of CRC and proposed a viable approach to treat late-stage metastatic CRC patients. LncRNA SNHG1 expression is upregulated in human CRC tissues. In the cytoplasm, SNHG1 sponges miR-154-5p, thereby reducing its ability to inhibit the expression of cyclin D2 (CCND2). In the nucleus, SNHG1 directly interacts with polycomb repressive complex 2 (PRC2) and modulates histone methylation at the promoters of Kruppel-like factor 2 (KLF2) and cyclin-dependent kinase inhibitor 2B (CDKN2B) (102). *In vivo* and *in vitro* experiments by Zhuang et al. (103) showed that lncRNA MALAT1 promotes CRC metastasis mainly *via* the lncRNA MALAT1/miR-106b-5p/SLAIN2 axis. LncRNA TTTY15 expression is abnormally upregulated in CRC tissues and it functions as a ceRNA by sponging miR-29a-3p to regulate the expression of the target gene DVL3, which affects the proliferation and metastasis of CRC (104). The results of *in vivo* and *in vitro* experiments have shown that a novel oncogenic lncRNA, RP11-757G1.5, which is overexpressed in CRC tissues, regulates the expression of YAP1 by sponging miR-139-5p and inhibiting its activity, thereby promoting the metastasis and invasion in CRC (105).

### LncRNAs Regulate CRC Metastasis Through Protein Binding

**Figure 2:**
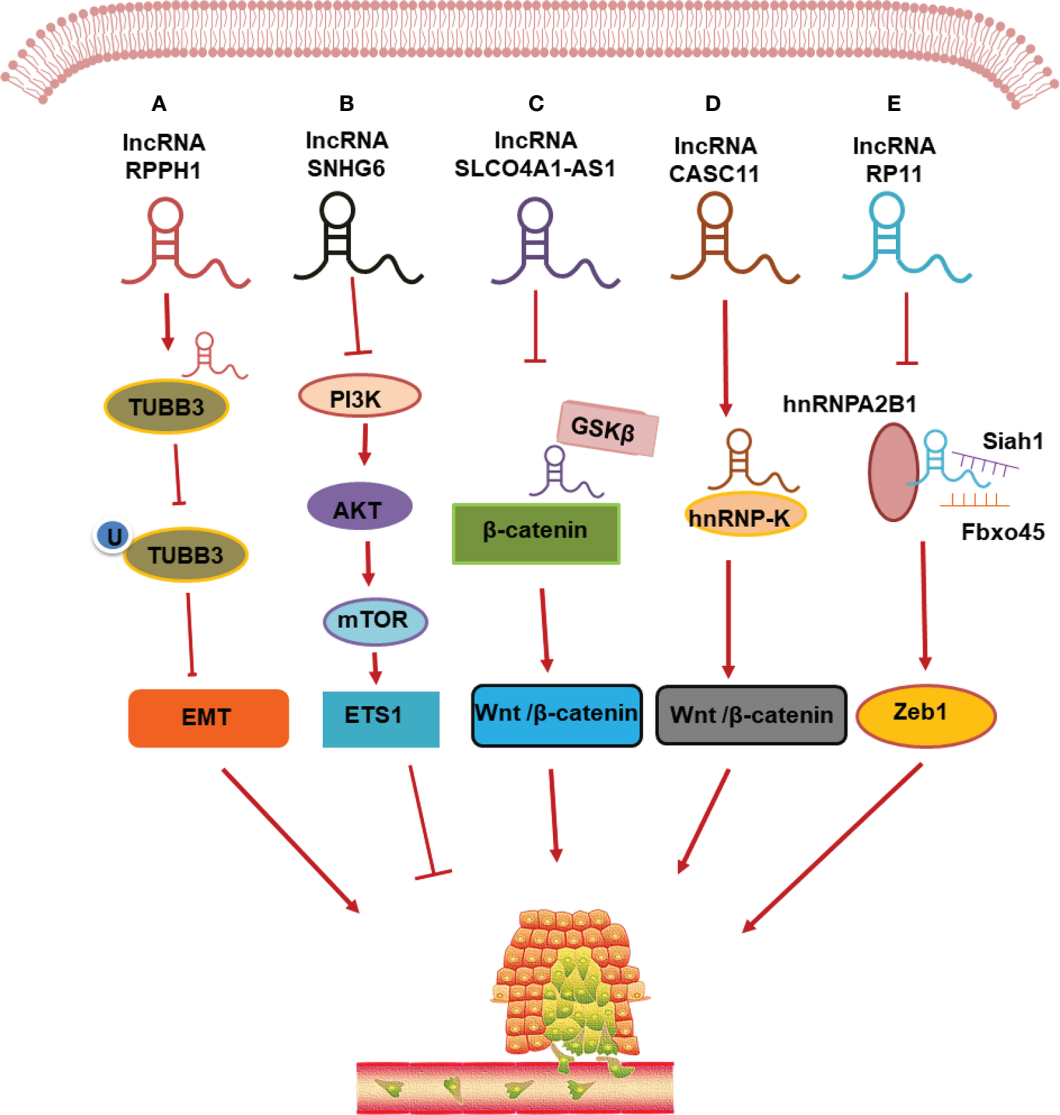
LncRNAs regulate CRC metastasis through protein binding. **(A)** lncRNA RPPH1 interacts with β-III tubulin (TUBB3) to prevent its ubiquitination and induces epithelial-mesenchymal transformation (EMT) of CRC; **(B)** lncRNA SNHG6 activates the endogenous colorectal cancer invasion pathway by down-regulating the expression of phosphoinositol 3-kinase (PI3K)/protein kinase B (AKT)/rapamycin mechanical target (mTOR); **(C)** lncRNA SlCO4a1-AS1 stabilized β-catenin by impairing the interaction of β-catenin with GSKβ, thereby activating Wnt/β-catenin signaling in CRC cells; **(D)** lncRNA CASC11 promotes CRC cell proliferation and metastasis by interacting with hnRNP-K protein and activating the WNT/β-catenin signaling; **(E)** lncRNA RP11 is involved in the CRC development by forming the RP11/hnRNPA2B1/mRNA complex, which accelerates the mRNA degradation of two E3 ligases Siah1 and Fbxo45 and prevents the proteasomal degradation of Zeb1 to increase its nuclear accumulation.

Similar to molecular chaperones, lncRNAs bind directly to transcription factors and form RNA-protein-DNA ternary complexes that regulate the transcription of downstream target genes involved in the CRC metastasis (**Figure 2**). LncRNAs act by two main mechanisms, which occur in different parts of the cells. In the nucleus, lncRNAs can coordinate with or antagonize transcription factors, thereby regulating the transcription of metastasis-related genes. In the cytoplasm, lncRNAs can bind to proteins and alter their post-translational modifications to induce the protein degradation; when these proteins are relevant to cancer, these effects can impact tumor metastasis.

The lncRNA SATB2-AS1 is specifically downregulated in CRC tissues. A mechanistic analysis showed that SATB2-AS1 binds directly to WDR5 and GADD45A and cis-activates SATB2 transcription by modulating histone H3 lysine 4 trimethylation (H3K4me3) and DNA demethylation in the SATB2 promoter region (106). A study by Wu et al. (107) showed that in intestinal cancer cells, the lncRNA RP11/hnRNPA2B1 (protein)/mRNA complex accelerated the degradation of Siah1 and Fbxo45 mRNAs, both of which encode ubiquitin E3 ligases, thereby preventing the proteasomal degradation of Zeb1, a transcription factor associated with EMT. This post-translational upregulation of Zeb1 is critical to RP11-induced dissemination of intestinal cancer cells. The lncRNA CPS1-IT can block hypoxia-induced autophagy by inhibiting HIF-1α levels, thereby preventing EMT and metastasis in CRC (108). Recent studies have shown that lncRNA RPPH1 can interact with β-III tubulin (TUBB3) to prevent its ubiquitination, which induces EMT and promotes CRC metastasis (109). The lncRNA LUCAT1 was shown to promote the proliferation, apoptosis, migration, and invasion of CRC cells *in vitro* and *in vivo*. Analysis showed that LUCAT1 binds to UBA52, which encodes ubiquitin, and the 60S ribosomal protein L40 (RPL40). By binding to UBA52, LUCAT1 targets the ribosomal protein L40/MDM2/p53 pathway to promote tumorigenesis and induce CRC cell cycle arrest and apoptosis (78). The lncRNA SNHG14, which is highly expressed in CRC, promotes CRC cell proliferation, motility, and EMT *in vitro*. SNHG14 promotes CRC progression by inhibiting EPHA7-mediated negative regulation through a process dependent on the transcription factor EZH2. SNHG14 enhances the stability of EZH2 mRNA by interacting with the RNA-binding protein FUS and sponging miR-186-5p, thereby mitigating miR-186-5p-induced silencing and increasing EZH2 expression in CRC (110). Ding et al. (111) found that the combination of lncRNA CRNDE and EZH2, a key component of PRC2, inhibited the expression of two downstream target genes dual-specific phosphatase 5 (DUSP5) and CDKN1A, which play important roles in CRC proliferation and metastasis. LINC01413 binds to hnRNP-K and induces nuclear translocation of YAP1 (associated protein 1) TAZ, thus regulating the expression of ZEB1 in CRC cells and promoting cancer metastasis (112). Zhang et al. (54) found that upregulation of lncRNA CASC11 in CRC is correlated with CRC growth and metastasis and that it exerts its effects by interacting with hnRNP-K protein and activating the Wnt/β-catenin pathway. Studies have shown that LINC01354 overexpression in CRC results in the enrichment of genes related to the Wnt/β-catenin signaling pathway. In CRC, LINC01354 mainly interacts with hnRNP-D to regulate the stability of β-catenin mRNA and activate the Wnt/β-catenin signaling pathway (50). The lncRNA ROR is a newly discovered lncRNA and Li et al. (113) demonstrated that knockout of the lncRNA ROR gene significantly increased the protein levels of p53 and its target genes, whereas the overexpression of ROR exerted the opposite effect. Thus, we conclude that the level of p53 protein is negatively correlated with ROR, and ROR may participate in the CRC progression *via* the p53 signaling pathway.

## Clinical Significance of lncRNAs in CRC Metastasis

Several studies have revealed that lncRNAs exert important biological effects in the CRC metastasis. Thus, the most practical application of lncRNAs is that they can be used as markers for early diagnosis of CRC metastasis. To improve the convenience and speed of CRC diagnosis, the differentially expressed lncRNAs can be detected in metastatic and non-metastatic samples (such as blood or urine). In addition, some lncRNAs closely correlate with the sensitivity to radiotherapy and chemotherapy, which may help to design novel therapies with better efficacy for the clinical treatment of metastatic CRC.

One challenge associated with existing diagnostic biomarkers of CRC is that they lack sufficient sensitivity and specificity, which can lead to false positive or false negative results. In recent years, several studies have shown that some lncRNAs can be detected in the blood, urine, serum, and other body fluids of patients with cancer (114). These lncRNAs could be used as biomarkers for the early diagnosis of cancer and prediction of patient prognosis (**Table 2**) (39, 48, 54, 59, 70, 74, 77, 78, 82, 84, 87, 88, 91, 94, 98–221). For example, lncRNA RP11-296E3.2, which is highly expressed in metastatic CRC, is associated with short overall survival (OS). In terms of its sensitivity and specificity of diagnosing CRC metastasis, RP11-296E3.2 was superior to CEA in plasma (113). Xu et al. (222) found that the plasma levels of four lncRNAs, ZFAS1, SNHG11, LINC00909, and LINC00654, were significantly lower in postoperative CRC samples than in preoperative samples. The combination of these four lncRNAs showed high diagnostic performance for early CRC. Studies have shown that lncRNA TINCR can affect the PI3K/Akt/mTOR signaling pathway by sponging miR-7-5p and playing a role in promoting CRC. In addition, compared with healthy controls, plasma levels of lncRNA TINCR were significantly elevated in CRC patients, which suggests its potential for the detecting early CRC (154). A correlation analysis by Pan et al. (223) showed that in patients with early CRC, plasma levels of lncRNA PVT1 are significantly higher than those of CEA, suggesting that PVT1 has great potential as a marker for the diagnosis of early CRC. A decrease in lncRNA-ATB expression significantly affects the progression of colon cancer by altering the expression of epithelial markers such as E-cad. A related clinical analysis showed that the level of plasma lncRNA-ATB was significantly increased in colon cancer patients at 1 month after surgery, suggesting that it may be useful for the early diagnosis of CRC (213). Ye et al. (80) observed that the level of lnc-GNAT1-1 in the plasma of CRC patients is related to tumor node metastasis (TNM) staging, while the receiver operating characteristic curve (ROC) showed that plasma lnc-GNAT1-1 has a moderate to good diagnostic efficiency for CRC.

**Table 2:** The correlation between LncRNAs and clinicopathological features in CRC.

LncRNA	Sample sources	Dysregulation	Relationship with clinicopathology	Ref.
LEF1-AS1	tissue	up	histological grade, lymph nodes metastasis	(115)
	tissue, plasma	up	tumor metastasis	(116)
SNHG1	tissue	up	tumor invasion depth, distant metastasis and TNM stage	(102)
	tissue	up	tumor diameter, TNM stage, lymph node metastasis, deep invasion	(117)
SNHG3	tissue	up	advanced clinical stage, distant metastasis	(118)
SNHG6	tissue	up	tumor stage, distant metastasis and lymph node metastasis	(119)
	tissue	up	size, TNM stage, and distant metastasis	(120)
CRNDE	serum	up	differentiation, invasion depth, lymph node metastases	(121)
SNHG7	tissue	up	clinical stage, lymph node metastasis, and distant metastasis	(94)
	tissue	up	size, lymphatic metastasis, distant metastasis and stage	(60)
SNHG15	tissue	up	lymph-node metastasis and liver metastasis	(122)
SNHG17	tissue	up	tumor size, TNM stage, and lymph node metastasis	(123)
B3GALT5-AS1	serum	down	tumor node metastasis stage and histological differentiation	(124)
	tissue	down	size, distant metastasis, and AJCC stages	(125)
Lnc-CMPK2	tissue	up	clinical stages and lymphatic metastasis	(56)
AGAP2-AS1	tissue	up	tumor stage	(126)
MIR4435-2HG	tissue	up	size and tumor stage	(98)
	tissue	up	size, lymph node metastasis, and tumor node metastasis staging	(127)
LDLRAD4-AS1	tissue	up	size, lymph node metastasis, TNM stage and vascular invasion	(128)
HOTAIR	tissue	up	venous invasion, tumor infiltration and distant metastasis	(129)
	tissue	down	invasion, lymph node, and organ metastasis, histological differentiation, vascular invasion, and tumor stage	(130)
LINC00152	tissue	up	size, grade, node metastasis (TNM) stage, and distant metastasis	(39)
LINC01413	tissue	up	size, stage, lymph node metastasis, and distant metastasis	(112)
SLCO4A1-AS1	tissue	up	local invasion and the TNM stage	(91)
ZEB1-AS1	tissue	up	clinical stage, lymph node metastasis, and distant metastasis	(131)
UCA1	tissue	up	lymph node metastasis, distant metastasis, and tumor stage	(132)
	tissue	up	differentiation, lymph node, and distant metastasis, invasion depth and size	(133)
DSCAM-AS1	tissue	up	lymphatic invasion	(134)
cCSC1	tissue	up	TNM stage, lymph node metastasis, and T stage	(78)
LINC01234	tissue	up	tumor stage, tumor size, and metastasis	(135)
LINC00460	tissue	up	TNM stage, T stage, and lymph node status	(136)
	tissue	up	clinical stage, M classification, N classification and liver metastasis	(137)
	tissue	up	depth of invasion and earlier pathological stages	(138)
	tissue	up	tumor size, tumor stage and lymph node metastasis	(139)
CCEPR	tissue	up	differentiation, clinical stage, lymph node metastasis, and distant metastasis	(140)
MIAT	tissue	up	lymph node metastasis and histologic grading	(141)
SATB2-AS1	tissue	down	invasion depth, TNM stage, lymph node, and distant metastasis	(106)
XIST	tissue	up	size, N1, M1, and TNM III+IV stage	(142)
BLACAT2	tissue	up	size, and lymph node (N), metastasis (M) and tumor-NM stages	(143)
DDX11-AS1	tissue	up	lymph nodes metastasis and TNM stage	(144)
CASC9	tissue	up	TNM stage	(70)
H19	tissue	up	tumor grade and metastasis	(145)
	tissue	up	low-grade differentiation and lymph node metastasis	(146)
CYTOR	tissue	up	TNM stage, T stage, N stage, and perineural and venous invasions	(147)
LINC01555	tissue	up	tumor stage	(148)
CASC19	tissue	up	liver metastasis, lymphatic metastasis, and TNM stage	(149)
LINC01354	tissue	up	tumor size, lymph metastasis, TNM stage, and distant metastasis	(50)
LINC00858	tissue	up	histological grade, lymph nodes metastasis, and TNM stage	(150)
SBF2-AS1	tissue	up	tumor size, TNM stage and lymph node metastasis	(151)
KAT7	tissue	up	site, size, differentiation, and lymph node metastasis	(152)
SATB2	tissue	down	T stage, lymph node metastasis and distant metastasis	(153)
TINCR	tissue	up	lymph node metastasis, differentiation and TNM stage	(154)
HOXD-AS1	tissue	down	differentiation and TNM stage	(74)
GIHCG	tissue	up	lymphovascular invasion, TNM stages, lymph node and distant metastasis	(155)
LUCAT1	tissue	up	TNM stage	(78)
LOC101927746	tissue	up	stage and metastasis	(156)
ST3Gal6-AS1	tissue	up	size, lymphatic metastasis, distant metastasis and stage	(87)
LINC00483	tisssue	up	clinical stage, M classification, N classification and liver metastasis	(157)
LncBRM	tissue	up	metastasis and stage	(158)
HULC	tissue	up	stage, size, and metastasis	(159)
APC1	tissue	down	clinical stage, lymph node and/or distant metastasis	(160)
ENST00000455974	tissue	up	TNM stage and distant metastasis	(161)
EWSAT1	tissue	up	depth of invasion, lymph node metastasis and TNM stage	(162)
LINC00657	tissue	down	tumor size and TNM stage	(163)
HOTTIP	tissue	up	tumor size, pathological stage, and distant metastasis	(164)
HOTTIP	tissue	up	T stage, clinical stage, and distant metastasis	(165)
SLCO4A1-AS1	tissue	up	size, lymph node metastasis, and TNM	(82)
Kcna3	tissue	down	TNM grade, lymphatic metastasis, and distant metastasis	(166)
HAND2-AS1	tissue	down	metastasis and stage	(167)
GAS5	tissue	down	clinical stage and lymph node metastasis	(168)
	tissues, plasma and exosomes	up	TNM stage, Dukes stage, lymph node metastasis (LNM), local recurrence rate, and distant metastasis rate	(169)
	tissue	down	tumor diameter and tumor-node-metastasis stage	(170)
	tissue	down	tumor size and TNM staging	(171)
SPINT1-AS1	tissue	up	regional lymph node metastasis and distant metastasis	(172)
u50535	tissue	up	lymph node metastasis and TNM stage	(173)
CASC15	tissue	up	clinical Tumor−Node−Metastasis stage and tumor metastasis	(174)
FTX	tissue	up	tumor diameter, TNM stage, lymph node, and distant metastasis	(99)
FEZF1-AS1	tissue	up	lymphatic invasion and tumor stage	(88)
LUADT1	tissue	up	size, metastasis, and TNM staging	(175)
HOXD−AS1	tissue	up	differentiation, distant metastasis, and TNM stage	(176)
DANCR	tissue	up	clinical stage, N classification, M classification, and liver metastasis	(177)
	tissue	up	TNM stage, histologic grade, and lymph node metastasis	(178)
AK098783	tissue	up	distant metastasis	(179)
PVT1	tissue	up	lymph node metastasis and tumor stage	(180)
	tissue	up	differentiation, depth, stage, node, TNM, and lymphatic metastasis	(181)
	tissue	up	distant metastasis	(182)
LncRNA00673	tissue	up	tumor, TNM stage, lymph node metastasis, distant metastasis, and size	(183)
XLOC_010588	tissue	up	sex, T-stage, and lymph node metastasis	(184)
DLEU7-AS1	tissue	up	tumor stage, lymph node metastasis, and distant metastasis	(185)
LncTCF7	tissue	up	tumor size, lymph node metastasis, and TNM stage	(186)
MALAT1	tissue	up	TNM stage	(187)
LINC00959	tissue	down	TNM stage, distant metastasis, and lymphatic metastasis	(188)
GHRLOS	tissue	down	lymph node metastasis and distant metastasis	(189)
CCAT2	tissue	up	differentiation, tumor infiltration, lymph node metastasis, distance metastasis, vascular invasion, and TNM stage	(190)
ZFAS1	tissue	up	Helicobacter pylori infection, lymph nodes metastasis and TNM stage	(191)
	tissue	up	lymphatic invasion and TNM stage	(192)
BANCR	tissue	up	lymph node metastasis	(193)
ZEB1-AS1	tissue	up	size, differentiation, TNM grade, depth of invasion, and Dukes’ classification	(194)
BC032913	tissue	down	lymph node and distant metastases	(195)
PlncRNA-1	tissue	up	depth of invasion, lymph node metastasis, and TNM stage	(84)
CRNDE	tissue	up	TNM stage	(77)
LINC01133	tissue	down	lymph node metastasis, distant metastasis, N classification, and TNM stage	(196)
UCC	tissue	up	lymph node metastasis and Dukes’ stage	(197)
linc-UBC1	tissue	up	size, tumor depth, lymph node metastasis, and TNM stages	(198)
PANDAR	tissue	up	local invasion, lymph node metastasis and TNM stage	(199)
PANDAR	tissue	up	diameter, histological differentiation, TNM stage, and depth of invasion	(200)
NNT-AS1	tissue	up	lymph node metastasis, TNM stage, vessel invasion, and differentiation	(201)
CCAL	tissue	up	TNM stage and metastasis	(202)
CRNDE-h	exosomes	up	regional lymph node metastasis and distant metastasis	(203)
	tissue	up	tumor size, regional lymph node metastasis, and distant metastasis	(204)
Loc554202	tissue	down	TNM stage, histologic grade, and lymph node metastasis	(205)
SPRY4-IT1	tissue	up	TNM stage	(206)
ANRIL	tissue	up	TNM staging, Duke staging and lymphatic metastasis	(207)
AFAP1-AS1	tissue	up	tumor size, TNM stage and distant metastasis	(208)
GAPLINC	tissue	up	larger tumor size, advanced tumor stage, and advanced node stage	(209)
lincRNA-ROR	tissue	up	T stage, N stage, AJCC stage, and vascular invasion	(210)
TUG1	tissue	up	grade, depth of tumor, lymph node-metastasis and liver metastasis	(211)
FEZF1	tissue	up	T-stage, lymph node metastasis, and distant metastasis	(212)
ATB	tissue	up	N stage and American Joint Committee on Cancer stage	(213)
	tissue	up	size, depth of invasion, lymphatic and invasion, and lymph node metastasis	(214)
NEAT1	tissue	up	tumor differentiation, invasion, metastasis and TNM stage	(215)
FER1L4	tissue	down	depth of invasion, lymph node metastasis, vascular invasion, and clinical stage	(216)
CLMAT3	tissue	up	liver metastasis and lymph node metastasis	(217)
MEG3	tissue	down	histological grade, deep tumor invasion, and TNM stage	(218)
RP11-462C24.1	tissue	down	distant metastasis	(219)
LOC285194	tissue	down	tumor size, tumor stage, and distant metastasis	(220)
PCAT-1	tissue	up	distant metastasis	(221)

LncRNAs have been shown to play roles in lymph node metastasis, lung metastasis, bone metastasis, and brain metastasis associated with several cancers (224). LncRNA CCAT2 is highly expressed in CRC and its expression is closely related to TNM stage as CCAT2 levels are increased from stages I to IV. High CCAT2 expression is closely associated with poor cell differentiation and depth of tumor invasion, lymph node metastasis, distant metastasis, vascular infiltration, and advanced TNM staging, and may be associated with increased liver metastasis (190). LINC00858 expression levels are significantly higher in CRC tissues than in adjacent tissues, and high LINC00858 expression is related to TNM staging, lymph node metastasis, and histological grade. Silencing of LINC00858 inhibits the proliferation, migration, and invasion of CRC cells and induces apoptosis (150). The expression level of MFI2-AS1 are closely related to tumor histological grade, lymphatic and distant metastasis, TNM staging, and vascular infiltration (225). High expression of lncRNA BANCR in CRC is associated with lymph node metastasis and the OS of patients with high BANCR expression is shorter (76). Chen et al. (226) divided 115 CRC patients into two groups based on the median lncRNA XIST expression level and an analysis of these groups showed that XIST expression was closely correlated with tumor size, histological grade, distant metastasis, and TNM staging. Similarly, the expression of lncRNA SNHG3 was significantly upregulated in CRC tissues, and SNHG3 expression was positively correlated with the advanced clinical stage and distant metastasis (118).

LncRNAs are an important group of molecules in the human transcriptome. LncRNAs play important roles not only in several physiological processes but also in various disease processes including cancer development and metastasis. Many lncRNAs are tumor specific and their expression can alter sensitivity to radiotherapy and chemotherapy. Therefore, they are expected to be useful as new therapeutic targets (227). LncRNA MALAT1, which was first found to be differentially expressed in patients with non-small cell lung cancer, is also significantly overexpressed in CRC. Low MALAT1 expression can inhibit the progression and metastasis of CRC and increase the sensitivity of cancer cells to 5-FU. This provides a new direction for designing novel therapeutic regimens for metastatic CRC (228). In addition, MALAT1 was found to be significantly upregulated in CRC tissues and cells treated with oxaliplatin. It promotes anti-oxidative response mainly *via* the miR-324-3p/ADAM17 axis and enhances sensitivity to oxaliplatin (229). In an experiment designed to select lncRNAs related to oxaliplatin resistance, Sun et al. (230) observed that the lncRNAs CRNDE, H19, UCA1, and HOTAIR affect the sensitivity to oxaliplatin. High expression of HOTAIR is associated with advanced tumor nodules and metastatic stages and poor prognosis of CRC. Peng et al. (231) observed that downregulation of lncRNA POU5F1P4 reduced the sensitivity of metastatic CRC cells to cetuximab, and could be a potential new treatment for metastatic CRC. Wang et al. (232) showed that the LINC00473 expression level was significantly higher in a group of drug-resistant patients than that in non-drug-resistant patients and knockdown of LINC00473 restored paclitaxel-induced cytotoxicity, inhibited cell viability and colony formation, induced apoptosis, and weakened the ability of tumor cells to migrate or invade.

## Discussion

The CRC metastasis is induced by a variety of factors *in vivo* and *in vitro.* Among the *in vivo* factors, changes in the tumor cell adhesion to surrounding cells and extracellular matrix, EMT, and the dysregulation of various motor proteins promote the CRC metastasis. Several signaling pathways such as Wnt/β-Catenin and PI3K/AKT signaling pathway play important roles in the CRC metastasis. LncRNAs also act as ceRNA to regulate the expression of downstream target genes or components of CRC metastasis-associated signaling pathways to impact CRC metastasis. Epidemiological studies have shown that CRC metastasis is closely related to several *in vitro* factors. For example, tea polyphenols (TPs) can exert anti-inflammatory, anti-oxidant, or pro-oxidant effects to promote apoptosis and act at multiple levels to inhibit CRC growth and metastasis (233). Nicotine upregulates the expression of UCA1 and HIF-1α in CRC cells and promotes the proliferation and metastasis of CRC cells (234). In addition, individuals with a family history of colorectal cancer and inflammatory bowel disease are more likely to develop colorectal cancer than individuals without such a family history of these diseases (4). Exploring the relationship among diet, lifestyle, and the risk of CRC occurrence and metastasis from the perspective of molecular epidemiology, and clarifying the critical exposure duration will help us better understand how these factors affect CRC occurrence and pathogenesis. Understanding the occurrence and development of the disease can help further to understand the clinical outcome (235). Elucidating the effects of *in vivo* factors, exploring the mechanism specific to colorectal cancer metastasis, identifying the important molecules involved in CRC pathogenesis will help the early clinical diagnosis and optimal treatment of CRC patients.

Few methods are available for CRC screening and most of the biomarkers used to diagnose CRC, such as CA199, are differentially expressed in many cancers. Therefore, CRC diagnosis lacks specificity and sensitivity. Mounting evidence has shown that abnormal expression of lncRNAs in human tissues and serum holds potential for early diagnosis and predicting patient prognosis. For example, expression of DANCR was lower in serum samples of postoperative patients than in patients with recurrence; moreover, serum DANCR expression significantly correlated with TNM staging (236).

Research has significantly advanced our understanding of the mechanisms underlying CRC and the therapeutic outcomes have been improved significantly. However, in metastatic CRC, the treatment outcomes, and mortality rate remain unsatisfactory. Therefore, there is an urgent need to find new therapeutic targets for metastatic CRC. Animal-based studies have shown that lncRNAs play important roles in metastatic CRC and can be used as potential targets for clinical treatment. Upon lncRNA-RI silencing, CRC cells show stronger radiosensitivity, making it a potential therapeutic target for metastatic CRC (237). Wu et al. (238) established a mouse xenograft model and observed that loss of lncRNA PVT1 and overexpression of miR-16-5p can minimize tumor volume. Through the lncRNA PVT1-miR-16-5p/VEGFA/VEGFR1/AKT axis, lncRNA PVT1 is directly involved in the progression of CRC and is a potential target for CRC treatment. Animal experiments by Yao et al. (239) showed that MIR600HG can inhibit tumor formation. Compared with lncRNA MIR600HG alone, combination therapy with MIR600HG and oxaliplatin significantly inhibited CRC stem cell metastasis and tumor growth.

Although lncRNAs have shown great potential in clinical applications, following gaps remain in lncRNA research. 1) The specific mechanisms underlying the effects of various lncRNAs in CRC remain unclear, highlighting the need for further research on the occurrence and development of CRC. 2) In terms of their utility as CRC biomarkers, the heterogeneity of lncRNA expression may make it difficult to achieve an accurate diagnosis. 3) Only a few animal experiments have been carried out to confirm treatment outcomes. Thus, limited data make it difficult to confirm the reliability of lncRNAs as diagnostic and therapeutic markers. Therefore, it is imperative to further explore the relationships between lncRNAs and CRC so that a solid foundation can be laid for their future use in CRC diagnosis and treatment. Nonetheless, research on lncRNAs in human cancers is expected to lead to major breakthroughs in terms of early diagnosis, risk detection, and treatment in the near future.

## Author Contributions

ZL, HN, JZ, and CO designed/planned the study and wrote the paper. All authors participated in writing the paper. ZL, HN, JZ, and CO performed imaging analysis. All authors contributed to the article and approved the submitted version.

## Funding

This study was supported by the National Natural Science Foundation of China (81903032), the China Postdoctoral Science Foundation (2020M672520), the Youth Fund of Xiangya Hospital (2018Q011), and the Mittal Innovative Entrepreneurial Project of Central South University (XCX20190719).

## Conflict of Interest

The authors declare that the research was conducted in the absence of any commercial or financial relationships that could be construed as a potential conflict of interest.

The reviewer QL declared a shared affiliation, with no collaboration, with several of the authors ZL, HN, YW, JZ, CO to the handling editor at the time of the review.
